# Spike-based adenovirus vectored COVID-19 vaccine does not aggravate heart damage after ischemic injury in mice

**DOI:** 10.1038/s42003-022-03875-y

**Published:** 2022-09-02

**Authors:** Shanshan Gu, Zhongyan Chen, Xiangfu Meng, Ge Liu, He Xu, Liying Huang, Linwei Wu, Jixing Gong, Ding Chen, Bingqing Xue, Lihang Zhu, Zhongjun Wan, Jianqing Lin, Xiaolong Cai, Xiaoyan Zhang, Jia Wang, Donghui Zhang, Nan Cao

**Affiliations:** 1 Zhongshan School of Medicine and the Seventh Affiliated Hospital, Sun Yat-Sen University, Guangdong, 510080 China; 2 Key Laboratory for Stem Cells and Tissue Engineering (Sun Yat-Sen University), Ministry of Education, Guangdong, 510080 China; 3 State Key Laboratory of Biocatalysis and Enzyme Engineering, Hubei Province Key Laboratory of Biotechnology of Chinese Traditional Medicine, National & Local Joint Engineering Research Center of High-throughput Drug Screening Technology, Hubei University, Wuhan, 430062 China; 4 GeneMedi Suzhou Biotechnology Co., Ltd, Shanghai, China; 5 Hanbio Biotechnology Shanghai Co., Ltd, Shanghai, China

**Keywords:** Cardiovascular diseases, Apoptosis

## Abstract

An unprecedented number of COVID-19 vaccination campaign are under way worldwide. The spike protein of SARS-CoV-2, which majorly binds to the host receptor angiotensin converting enzyme 2 (ACE2) for cell entry, is used by most of the vaccine as antigen. ACE2 is highly expressed in the heart and has been reported to be protective in multiple organs. Interaction of spike with ACE2 is known to reduce ACE2 expression and affect ACE2-mediated signal transduction. However, whether a spike-encoding vaccine will aggravate myocardial damage after a heart attack via affecting ACE2 remains unclear. Here, we demonstrate that cardiac ACE2 is up-regulated and protective after myocardial ischemia/reperfusion (I/R). Infecting human cardiac cells or engineered heart tissues with a spike-based adenovirus type-5 vectored COVID-19 vaccine (AdSpike) does not affect their survival and function, whether subjected to hypoxia-reoxygenation injury or not. Furthermore, AdSpike vaccination does not aggravate heart damage in wild-type or humanized *ACE2* mice after I/R injury, even at a dose that is ten-fold higher as used in human. This study represents the first systematic evaluation of the safety of a leading COVID-19 vaccine under a disease context and may provide important information to ensure maximal protection from COVID-19 in patients with or at risk of heart diseases.

## Abstract (web summary)

The safety of spike-based adenovirus type-5 vectored COVID-19 vaccine is evaluated, showing it neither affects the survival and function of human cardiac cells and engineered heart tissues nor aggravates heart damage in mice after ischemic injury.

## Introduction

The ongoing pandemic of coronavirus disease 2019 (COVID-19) caused by a coronavirus SARS-CoV-2 has spurred an unprecedented public health crisis worldwide. Hence, the development of a safe and effective vaccine that can prevent SARS-CoV-2 infection and transmission has rapidly become top priority. For cell entry, the SARS-CoV-2 virus majorly binds to the host receptor angiotensin-converting enzyme 2 (ACE2) through its spike glycoprotein, which is the only viral protein that interacts with host cells and is the most diverging protein between different coronaviruses^1^. Therefore, generating a vaccine encoding/introducing the spike protein is the strategy used by the majority of COVID-19 vaccine candidates, including vaccines based on viral vectors, nanoparticles/virus-like particles, proteins/peptides, RNA, and DNA^2^. There has been an unprecedented rapid response by vaccine developers with now over 167 COVID-19 vaccine candidates in clinical trials and 15 having been approved for at least limited use as of 28 June 2022. Although trials of the approved spike-based COVID-19 vaccines have not detected vaccine-related serious adverse events, it should be noticed that safety of these vaccines was mostly evaluated in healthy volunteers, and little is known about their effects on patients with or at risk of chronic diseases^3–5^. To date, clinical study that evaluating the safety of COVID-19 vaccination in patients with preexisting ischemic heart diseases has not been reported. In addition, as worldwide vaccination goes on rapidly, the COVID-19 vaccine has been associated with an elevated risk of several cardiac adverse events (e.g., acute myocardial infarction and cardiac arrest) according to real-world safety data^6–9^. It is worth noting that vaccine-associated acute myocardial infarction and cardiac arrest are more common in individuals aged >75^6^, who are theoretically at high risk of cardiovascular diseases. Although the causal relationship is yet to be established, and the adverse events following vaccination may not be considered purely caused by the vaccines themselves, it raises concern about cardiac safety of the COVID-19 vaccine. Furthermore, whether the vaccination in individuals with cardiovascular comorbidities would exaggerate cardiac injury remains unclear, and warrants further experimental and clinical investigation.

ACE2 is a membrane-localized aminopeptidase that is highly expressed in the heart and blood vessels and has direct effects on multiple organs via counter-regulation of the renin-angiotensin system (RAS), a primary cardiovascular regulatory system^10^. Knockout of *ACE2* in mice resulted in a reduction in cardiac contractility^11^, indicating that ACE2 is an essential regulator of heart function. Moreover, organ-protective effect of ACE2 has been well-documented in hypertension, diabetes, atherosclerosis, and acute respiratory distress syndrome^12^. Increased ACE2 expression is observed after myocardial infarction in both rodents and human^13^, suggesting that ACE2 may also protect the heart following ischemic injury. However, interaction of the spike protein with ACE2 during virus infection has been shown to reduce ACE2 expression^14,15^, and alters the RAS signal transduction. For example, binding of spike to ACE2 upregulates the Ras-ERK-AP-1 pathway leading to activation of pro-remodeling factors such as the C-C motif chemokine ligand 2, which may contribute to cardiac injury and subsequently cause the fibrosis associated with disease manifestation^16^. Hence, this raises a pivotal question: will the vaccine encoding the spike protein increase the risk of myocardial damage after a heart attack via binding to and affecting the cardiac ACE2? For patients with coronary or ischemic heart disease or people at high heart attack risk (e.g., arrhythmias, hypertension, or diabetes mellitus), questions arise around the safety after COVID-19 vaccinations in the setting of increased ACE2 expression in the injured heart and warrant close investigation. Thus, in this study, we evaluate the effects of spike-expressing vaccine on hearts that are subjected to ischemia/reperfusion (I/R) injury using human cardiac cells, human-engineered heart tissues, and humanized *ACE2* (hACE2) mice.

## Results

### ACE2 is upregulated after myocardial I/R and limits heart injury

**Fig. 1:**
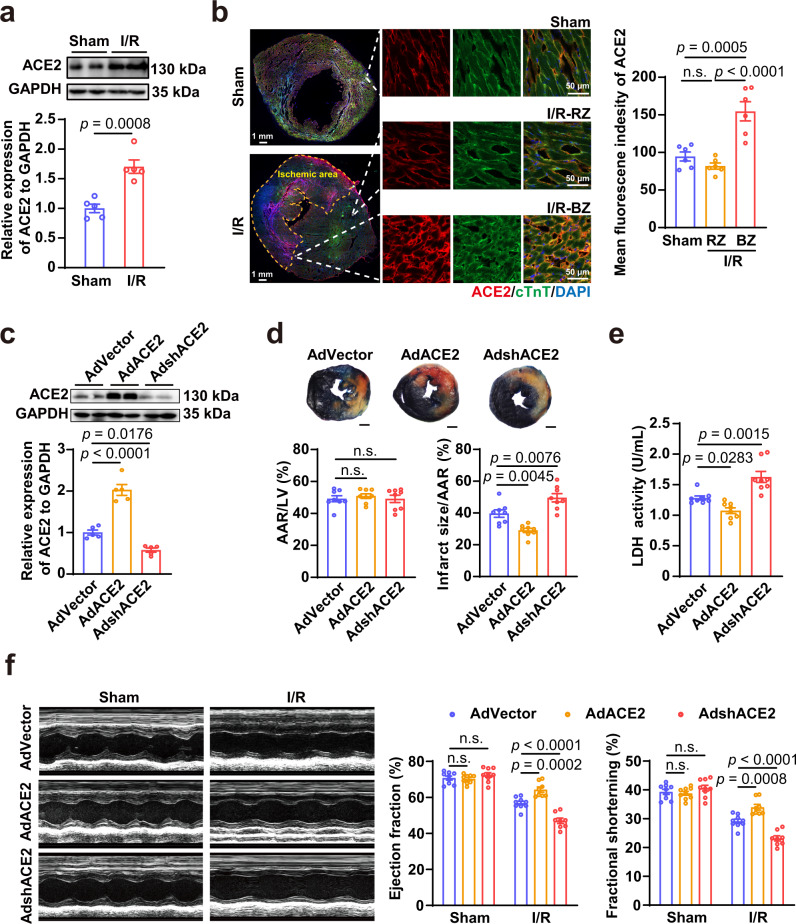
The protective effect of ACE2 during myocardial I/R injury. **a** Representative and quantitative immunoblot analysis of ACE2 protein in the hearts after myocardial I/R (45 min/24 h). *n* = 5 for each group. **b** Representative immunostaining analysis of ACE2 expression with quantification of the mean fluorescence intensity in the hearts after myocardial I/R (45 min/24 h). RZ, remote zone; BZ, border zone. *n* = 6 for each group. **c** Representative and quantitative immunoblot analysis of ACE2 protein in the hearts infected with adenovirus-mediated vector control (AdVector), adenovirus-mediated ACE2-overexpression (AdACE2), or adenovirus-mediated ACE2 short hairpin RNA (AdshACE2) for 3 days. *n* = 5 for each group. **d** Representative 2, 3, 5-triphenyltetrazolium chloride (TTC)/Evans blue staining (upper) and averaged infarct size (lower) following myocardial I/R (45 min/24 h). AAR, area at risk; LV, left ventricle; *n* = 8 for each group; Scale bar, 1 mm. **e** The lactic dehydrogenase (LDH) activity in the serum of mice infected with AdVector, AdACE2, or AdshACE2 following myocardial I/R (45 min/24 h). *n* = 8 for each group. **f** Representative M-mode echocardiogram images and statistical analysis of ejection fraction and fractional shortening in the hearts infected with AdVector, AdACE2, or AdshACE2 following myocardial I/R (45 min/24 h). *n* = 8–9 for each group. Statistical significance was assessed using unpaired, two-tailed Student *t* test (**a**), one-way ANOVA with Turkey post-test (**b**–**e**) or 2-way ANOVA with Turkey post-test (**f**). Data are presented as mean ± SEM; n.s., not significant.

To investigate the role of ACE2 in heart repair, we examined its expression in the injured mouse heart after myocardial I/R. We have confirmed the increase of ACE2 following injury (Fig. 1a), with a particular activation at the border zones of I/R hearts (Fig. 1b). Notably, we found that ACE2 overexpression reduced the infarct size and improved heart function during I/R, whereas ACE2 knockdown aggravated heart damage (Fig. 1c–f). These data suggest that ACE2 has a protective role during myocardial I/R. Hence, whether vaccines encoding the spike protein will aggravate myocardial damage after I/R via interfering ACE2 warrants serious investigation (Supplementary Fig. 1).

### Spike-based vaccine has little effect on cell survival of human cardiac cells with or without hypoxia-reoxygenation (hyp-reox) injury in vitro

**Fig. 2:**
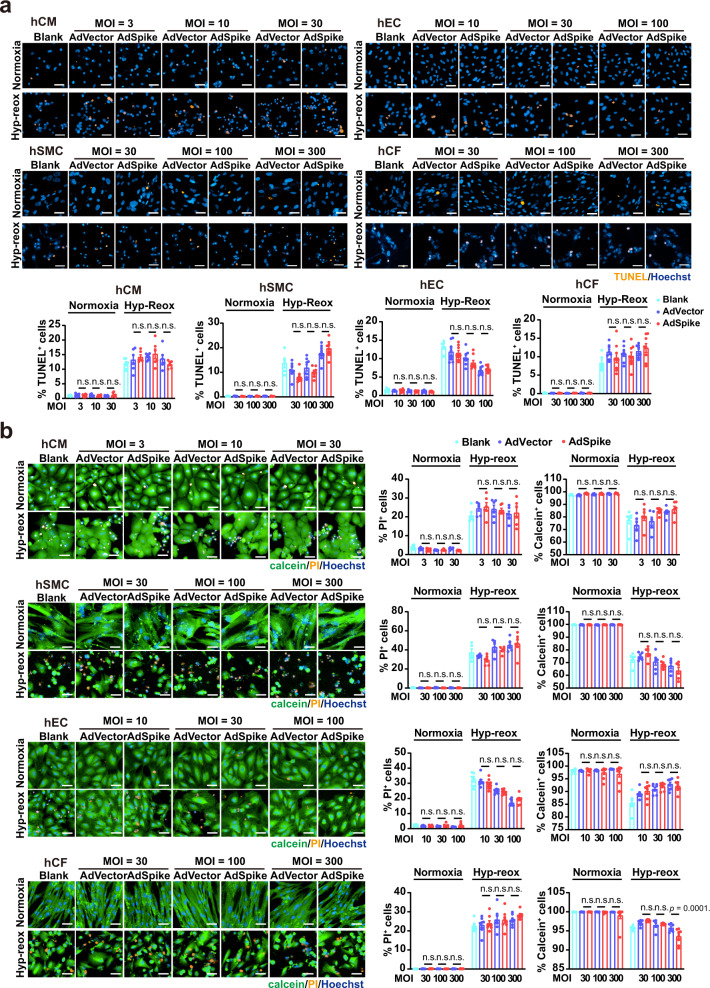
AdSpike has little effect on cell survival of human cardiac cells with or without hyp-reox injury. **a** Representative and quantitative immunostaining analysis of the TUNEL^+^ cells in hCMs, hSMCs, hECs, and hCFs infected by AdVector or AdSpike with or without hyp-reox injury. Scale bar, 50 μm. *n* = 5–8 for each group. **b** Representative and quantitative calcein-AM/propidium iodide (PI) double staining in hCMs, hSMCs, hECs, hCFs infected by AdVector or AdSpike with or without hyp-reox injury. *n* = 5–8 for each group. Statistical significance was assessed using 2-way ANOVA with Turkey post-test. Scale bar, 50 μm. Data are presented as mean ± SEM; n.s., not significant.

We firstly examined the ex vivo effect of the spike-based vaccine on the four major cell types of human heart (Supplementary Fig. 2a), using hESC-derived cardiomyocytes (hCMs), hESC-derived smooth muscle cells (hSMCs) (Supplementary Fig. 2b, c), primary human cardiac fibroblast (hCFs), and human endothelial cells (hECs). A recombinant adenovirus type-5 (Ad5) vectored COVID-19 vaccine expressing the spike protein (AdSpike) was chosen as a proof-of-principle, because it has the longest expression period when compared with the vaccines that temporally deliver the spike DNA, mRNA, or recombinant protein, thus may have a theoretically maximum effect on the host tissues and cells. We fine-tuned the multiplicity of infection (MOI; i.e., the number of infectious viral particles per cell) of AdSpike to achieve either low (50–70%), moderate (~90%), or high (~100%) expression efficiency of spike on each cell type (Supplementary Fig. 2d), and evaluated their effects on cell survival under normoxia condition or after hyp-reox injury. As expected, in all four cell types examined, normoxic cultured cells that received the control adenovirus (AdVector) or AdSpike had a comparable survival rate as revealed by the terminal-deoxynucleotidyl transferase-mediated nick end labeling (TUNEL) (Fig. 2a) and calcein-AM/propidium iodide staining (Fig. 2b) analyses. Notably, AdSpike had no or minimal influence on cell survival in hCMs, hSMCs, hCFs, or hECs that underwent hyp-reox (Fig. 2a, b). Similar observations were also seen in adult rat cardiomyocytes (arCMs) or neonatal rat cardiomyocytes (nrCMs) (Supplementary Figs. 2e and 3a, b). These data suggest that spike-based vaccine has little effect on the survival of cultured cardiovascular cells even after hyp-reox injury.

### AdSpike does not alter the calcium handling properties of cardiomyocytes cultured in normoxia or hyp-reox conditions

**Fig. 3:**
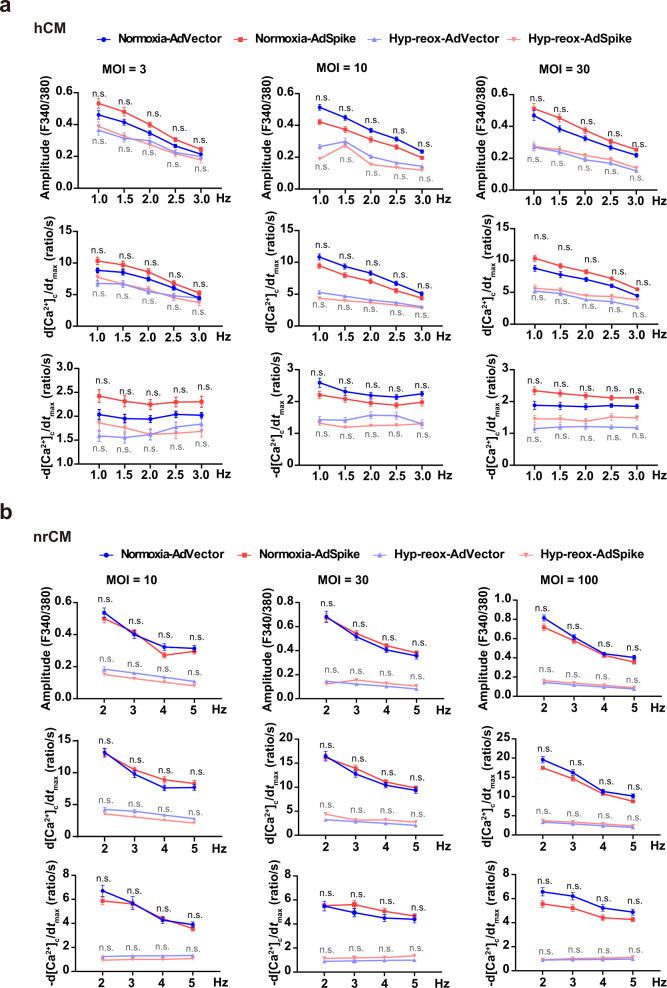
AdSpike has little effect on calcium handling properties of cultured cardiomyocytes subjecting to electrical field stimulation with or without hyp-reox injury. Averaged parameters of calcium transient of hCMs (**a**) or neonatal rat cardiomyocytes (nrCM, **b**) infected by AdVector or AdSpike with different frequency of electrical field stimulation. *n* = 15–30 for each group. Statistical significance was assessed using 2-way ANOVA with Turkey post-test. Data are presented as mean ± SEM; n.s., not significant as compared with the corresponding AdVector control, in which the black color indicates comparison within the normoxia group, and gray color indicates comparison within the hyp-reox group.

Dysfunction of calcium handling is one of the functional features in CMs that suffer from cytotoxicity or injury, and some toxins can disturb CM function without affecting cell death, especially for those with mild toxicity^17^. To explore whether AdSpike affects hCM function in both normoxia and hyp-reox situations, we assessed the calcium handling properties in hCMs that were infected by AdVector or AdSpike at various MOI. When subjected to a series of increasing electrical field stimulation frequencies, we found that AdSpike-infected hCMs could keep pace with the increasing stimulation under both normoxia and hyp-reox situations, similar to what was observed in the AdVector control. Moreover, no differences in the amplitude and rise/decay rates of Ca^2+^ transients were observed among the AdSpike groups and the AdVector groups with or without hyp-reox injury (Fig. 3a). This conclusion was independently confirmed in nrCMs (Fig. 3b). The harmonic response of AdSpike-infected cardiomyocytes indicates that their calcium handling machinery could take up and release calcium stores in time for higher pulse stimulation frequencies. Thus, spike-based vaccine does not affect the calcium handling properties of cultured cardiomyocyte with or without hyp-reox injury.

### AdSpike minimally affects the survival and function of human engineered heart tissues (hEHTs) with or without hyp-reox injury

**Fig. 4:**
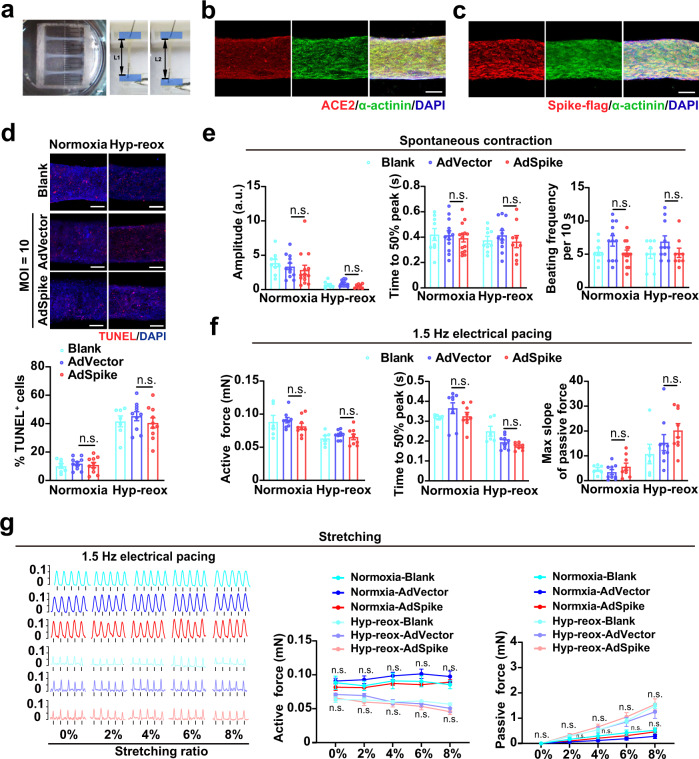
AdSpike has little effect on hEHTs that underwent spontaneous contraction, electrical field stimulation, and mechanical tensile test with or without hyp-reox injury. **a** Low magnification image of hEHT on day 21 (left) and the equipment image of mechanical contractility force test for hEHT (right). Scale bar, 2 mm. **b**, **c** Co-localization analysis of anti-α-actinin (green) and anti-ACE2 (red) or anti-Spike-flag (red) with DAPI (blue). Scale bar, 200 μm. **d** Representative image and quantification of TUNEL staining in hEHTs with or without hyp-reox injury. *n* = 7–10 for each group. Scale bar, 200 μm. **e** Video analysis of the hEHTs for spontaneous contraction amplitude, time to 50% peak, and beating frequency per 10 seconds. a.u., absolute units. *n* = 9–14 for each group. **f** The active contractile force, time to 50% peak and maximum slope of passive force of the bundles at 1.5 Hz electrical pacing with or without hyp-reox injury. *n* = 6–9 for each group. **g** Representative contractile force traces and active contractile force during progressive stretching (0%, 2%, 4%, 6%, and 8% tissue length) of hEHTs with or without hyp-reox injury on day 22. *n* = 5–9 for each group. Statistical significance was assessed using 2-way ANOVA with Turkey post-test. Data are presented as mean ± SEM; n.s., not significant, compared with the corresponding AdVector control, in which the black color indicates comparison within the normoxia group, and gray color indicates comparison within the hyp-reox group.

To provide a further assessment of AdSpike on human heart tissue with or without ischemic injury, we took advantage of hEHT model (Fig. 4a; Supplementary Fig. 4) which widely expressed ACE2 (Fig. 4b). We infected cells with AdSpike while fabricating hEHTs (Fig. 4c; Supplementary Fig. 4a). The infection efficiency was very high as indicated by staining of the spike-fused flag epitopes (Fig. 4c). Under both normoxia and hyp-reox conditions, we found that they exhibited similar cell apoptosis (Fig. 4d) and spontaneous contraction properties, including amplitude, 50% peak time, and beating rate (Fig. 4e). Consistently, there was no difference between the AdVector and AdSpike groups in active contractile force, time to 50% peak and maximum slope of passive force of the bundles at 0% stretch with or without hyp-reox injury under 1.5 Hz electrical pacing (Fig. 4f). More importantly, we used a customized contractility force test system to analyze the hEHTs’ contraction in stepped raising stretching length (stretching ratio 0%, 2%, 4%, 6%, 8%)^18^. We observed that the contractility force increased while stretching in both AdVector and AdSpike hEHT groups, whereas there was no difference in active force and passive force between the two groups in both conditions (Fig. 4g). In aggregate, the spike-encoding vaccine does not affect hEHT function in both normoxia and hyp-reox situations.

### AdSpike has little effect on heart damage repair after myocardial I/R in vivo

**Fig. 5:**
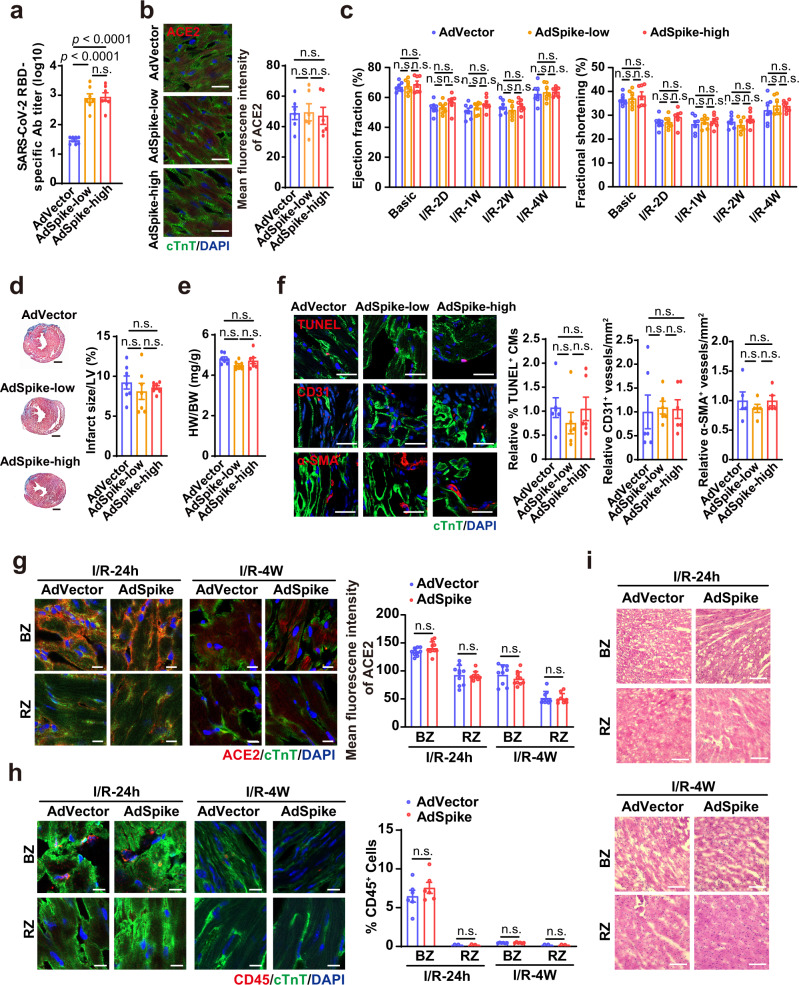
AdSpike has little effect on heart damage repair after myocardial I/R on hACE2 mice in vivo. **a** Antibody tilters of SARS-CoV-2 spike receptor-binding domain (RBD) in the mouse serum collected at 4 weeks after injection of AdSpike at various doses. *n* = 7 for each group. **b** Representative immunostaining analysis of the ACE2^+^ cardiomyocytes with quantification of the mean fluorescence intensity in the heart at 4 weeks post-vaccination. *n* = 6 for each group. Scale bar, 20 μm. **c** Ejection fraction and fractional shortening of hACE2 mice heart after I/R measured by echocardiography at various time points post-I/R measured by echocardiography. D, day; W, week. *n* = 7 for each group. **d** Masson-Trichrome staining of heart cross sections of hACE2 mice 4 weeks post-I/R (left) with quantification of scar size (right). *n* = 7 for each group. Scale bar, 1 mm. **e** Quantification of heart weight (HW) relative to body weight (BW) of hACE2 mice at 4 weeks post-I/R. *n* = 7 for each group. **f** Representative and quantitative immunostaining analysis of TUNEL^+^/cTnT^+^ cardiomyocytes, CD31^+^ capillaries, and α-SMA^+^ vessels in the border zones of infarcted hACE2 mouse hearts 4 weeks post-I/R. *n* = 5–6 for each group. Scale bar, 50 μm **g** Representative immunostaining analysis of the ACE2^+^ cardiomyocytes with quantification of the mean fluorescence intensity in border zone (BZ) or remote zone (RZ) at 24 hours or 4 weeks post-I/R. *n* = 9–10 for each group. Scale bar, 10 μm. **h** Representative and quantitative immunostaining analysis of CD45^+^ cells in border zone (BZ) or remote zone (RZ) at 24 hours or 4 weeks post-I/R. *n* = 6 for each group. Scale bar, 10 μm. **i** Representative hematoxylin-eosin staining analysis of border zone (BZ) or remote zone (RZ) at 24 hours or 4 weeks post-I/R. Scale bar, 100 μm. Statistical significance was assessed using one-way ANOVA with Turkey post-test (**a**, **b**, **d**–**f**) or 2-way ANOVA with Turkey post-test (**c**, **g** and **h**). Data are presented as mean ± SEM; n.s., not significant.

To further explore the in vivo effects of spike-based vaccine on hearts after myocardial I/R injury, we intramuscularly injected AdSpike into wild-type C57BL/6 mice at a dose that is equal (1 × 10^9^ viral particles per kilogram of body weight, AdSpike-low) or ten-fold higher (1 × 10^10^ viral particles per kilogram of body weight, AdSpike-high) as used in human^19^. Then the mice were subjected to myocardial I/R injury 7 days post-vaccination, and the effects of AdSpike on heart function were examined over a period of 28 days (Supplementary Fig. 5a). AdVector at a dose of 1 × 10^10^ viral particles per kilogram of body weight was used as a control. I/R injury was created by temporal ligation of the left anterior descending coronary artery in vivo for 1 hour followed by reperfusion. The spike protein (detected by examination of the spike-fused flag epitope) started to increase 7 days after vaccination, and stably expressed thereafter until day 14 (Supplementary Fig. 5b). Effective immunogenicity of AdSpike at both high and low doses was confirmed by the specific ELISA antibody responses to the receptor binding domain 28 days post-vaccination (Fig. 5a). By high-resolution echocardiography, we found that the heart function was equally preserved after AdSpike vaccination compared to the AdVector control, reflected by the left ventricular ejection fraction and fractional shortening (Supplementary Fig. 5c). Consistently, we observed comparable scar sizes and heart weight/body weights between the vaccinated and control mice (Supplementary Fig. 5d, e). These data suggest that spike-based vaccine does not aggravate heart damage in wild-type mice after myocardial I/R injury.

It is known that the orthologous ACE2 receptor in mice has a lower affinity to bind to the spike protein, therefore typical inbred mouse strains do not support robust SARS-CoV-2 infection and replication^20^. To exclude the possibility that the above observation was an experimental artifact caused by the inefficient binding between spike and the mouse ACE2, we used a humanized mouse model in which the human ACE2 coding sequence was knocked-in into the mouse *Ace2* genomic locus and replaced its mouse ortholog (Supplementary Fig. 5f, g). Following AdSpike vaccination, the expression of spike and its interaction with ACE2 in heart tissues of hACE2 mice remained almost undetectable (Supplementary Fig. 5h). Moreover, we observed that ACE2 expression was not declined at both doses (Fig. 5b). In accordance with the observation in wild-type mice, histological and functional influences of AdSpike vaccination on I/R hearts of hACE2 mice were neglectable when compared with the controls (Fig. 5c–e). Furthermore, neither the number of TUNEL^+^ apoptotic cardiomyocytes nor vascular density in the border zone was altered by AdSpike vaccination (Fig. 5f). Furthermore, we confirmed that ACE2 expression was not reduced by AdSpike vaccination at both 24 hours and 4 weeks after myocardial I/R (Fig. 5g). Since immune responses are critical mediators of tissue damage after myocardial infarction^21,22^, we examined the immune responses in the I/R injured heart tissues of the hACE2 mice after AdSpike vaccination. Once again, we found that the inflammatory cell infiltration was comparable between the vaccinated and the control groups at both 24 hours and 4 weeks after myocardial I/R (Fig. 5h, i). Taken together, spike-based vaccine has no effect on cardiomyocyte apoptosis, scar formation, infarct revascularization, and function recovery during myocardial I/R injury.

## Discussion

Hopes for a COVID-19 vaccine are now a reality. Although the dominant pathology of COVID-19 involves the respiratory system, 20–30% of COVID-19 patients experience severe cardiovascular damage, which emerged as a major indicator of poor prognosis^14,23^. Moreover, patients with pre-existing heart complications are more likely to develop severe illness and have higher risk of death compared with patients without co-morbidities^23–26^. Therefore, a COVID-19 vaccine would be lifesaving for patients with or at risk of heart diseases, which are considered a high-priority subgroup for COVID-19 vaccination. Limited data are available, however, on the safety, tolerability, and efficacy of COVID-19 vaccines in this group of people, owing to the exclusion of patients with heart diseases from most of the COVID-19 vaccination trials. Here, by combining models of human pluripotent stem cell-derived cardiac cells/tissues and humanized mouse models, we provide a proof-of-principle demonstration that spike-based Ad5 vaccine does not increase myocardial damage after ischemic injury. To our knowledge, this study represents the first systematic evaluation of the safety of a leading COVID-19 vaccine under a heart disease context.

Previous studies have demonstrated that ACE2 is downregulated upon interaction with the spike protein of SARS-CoV^15^ and SARS-CoV-2^14,27^. Several mechanisms have been identified or proposed to explain this phenotype. Firstly, upon interacting with the viral spike protein, ACE2 is internalized along with the virion and translocated into the endosomes of host cell, through which the ACE2 level on the cell membrane is reduced^28^. 2) Binding of spike protein to ACE2 activates metalloproteinase domain 17 (ADAM17), which cleaves the membrane-bound ACE2, resulting in the shedding of ACE2 extracellular domain and the reduction of membrane-bound ACE2^28^. 3) SARS-CoV infection of myocardial and lung tissues inhibits *Ace2* mRNA expression^15,29^. We hypothesize that vaccine-generated spike protein may get into the myocardium and reduce ACE2 level in the host cardiac cell membrane via one of the mechanisms mentioned above. In this study, we examined the expression of ACE2 in both healthy and I/R injured mouse hearts upon spike-based vaccination and found that it remained unchanged (Fig. 5b, g). Most likely the reason is that the amount of COVID-19 vaccine-generated spike protein is not abundant enough to get into myocardium and affects cardiac ACE2.

There are several limitations to this study: 1) only a specific myocardial I/R protocol, in which the injury is induced 7 days after vaccination, is used. Whether other timeline of I/R injury (e.g., I/R happened shortly before vaccination or concurrently with vaccination) would affect heart repair warrants further exploration; 2) only Ad5-vector vaccine is included and alternative types of COVID-19 vaccines that deliver the spike protein with various modifications should be examined similarly in the follow-up studies; 3) I/R is the only examined disease of heart injury and the animal number size is still relatively small. Similar evaluation of cardiac safety of the COVID-19 vaccines should be performed under other cardiovascular pathological conditions. Although there are still many unanswered questions, we expect the spike-based COVID-19 vaccine to be safe and at least partly effective after myocardial ischemic injury, and benefits of vaccination likely outweigh risks of vaccine-related adverse events. This study may not only pave the way for clinical trials of people with cardiovascular conditions receiving these vaccines, but also provide an example that may be adapted to elucidate the safety and efficacy of COVID-19 vaccines in other complicated diseases context.

## Methods

### Animal care

All mouse work was done in accordance with the university guidelines (Sun Yat-sen University Animal Care and Use Committee). The male C57BL/6 mice were purchased from the Laboratory Animal Center of Sun Yat-Sen University. The C57BL/6 background hACE2 mice (T037659) were purchased from Gempharmatech Co. Ltd (Nanjing, China). Mice aged at 10–12 weeks were used for experiments.

### Myocardial ischemia/reperfusion (I/R) model

The myocardial I/R model was induced as previously described^30^. Briefly, the adult male mice aged 10–12 weeks were anesthetized using pentobarbital sodium (50 mg/kg, i.p.) and ventilated using a rodent ventilator. Then we performed a left thoracotomy and ligated the left anterior descending (LAD) coronary artery in the left ventricle using sterile 7-0 silk sutures with a slipknot. For reperfusion, the ligation was removed at the end of ischemia. For long-term heart function analysis, the hearts were performed ischemia for 1 h followed by a 4-week reperfusion. The hearts were then harvested for scar area analysis using the Masson’s trichrome staining. For short term analysis during acute phage, the time of ischemia was 45 min followed by a 24-h reperfusion. The infarct size was analyzed by 2,3,5-triphenyltetrazolium chloride (TTC)/Evans blue staining. Briefly, the LAD was retied for the determination of area at risk with 5% Evans blue (E2129, Sigma) injected into the external iliac vein. Then the hearts were sliced for TTC staining (1% TTC, T8877, Sigma; 37 °C, 15 min) to visualize the unstained infarcted region. The scar area and infarct area were determined by planimetry with the ImageJ software.

### Cardiac function assessment by echocardiography

Mice were anesthetized with inhalation of isoflurane (1–1.5%) and examined by transthoracic echocardiography using a Vevo 3100 high-resolution imaging system with 40-MHz (VisualSonics Inc.). The long-axis views in M-model were obtained for calculating the left ventricle ejection fraction and fractional shortening.

### Construction of Ad5 vectored COVID-19 vaccine

An Ad5 vectored COVID-19 vaccine expressing the spike glycoprotein of SARS-CoV-2 (AdSpike) was prepared as described previously^19^. Briefly, an optimized full-length spike gene based on the Wuhan-Hu-1 (GenBank accession number YP_009724390) was fused with a flag epitope sequence, cloned and packaged to Ad5 adenovirus using the Admax system. Then the Ad5 vectored COVID-19 vaccine was purified and prepared as liquid formulation containing 2 × 10^10^ viral particles per mL before intramuscular injected into the mice.

### Construction of recombinant adenoviruses expressing ACE2 and the shRNA for *ACE2*

Recombinant adenoviruses (Ad) expressing human ACE2 (AdACE2) and short hairpin RNA of *ACE2* (AdshACE2) were prepared using a pAdEasy vector system (Qbiogene, USA) and homologous recombined in bacteria BJ5183. The recombinant plasmids were then transfected separately into HEK 293 cells for the package of adenovirus. The target sequence of AdshACE2 was: 5-CCGTAACCAGTTGATTGAAGATGTA-3.

### Gene delivery in the heart in vivo

The adenovirus was injected into the hearts as previously described^30^. Briefly, following the thoracotomy on mice anesthetized, the diluted adenoviruses (3 × 10^10^ pfu/ml, 30 μl) in a 30-gauge needle were injected into three sites of the left ventricle from the apex to the aortic root^30,31^. The expression analysis of the genes delivered into the heart was performed 3 days later. Then the hearts were subjected to myocardial I/R injury.

### Immunoblotting analysis

Myocardium tissues collected from the area at risk of the left ventricle were homogenized with RIPA lysis buffer including 20 mM Tris-HCl (pH 7.4), 1% Triton X-100, 1% deoxycholate, 10% glycerinum, 150 mM NaCl, 2.5 mM EDTA, and 1 mg/ml protease inhibitor cocktail. Then the tissue homogenates were analyzed by standard immunoblotting analysis with specific antibodies against ACE2 (1:1000, ab15348, Abcam) and GAPDH (1:8000, 97166, Cell Signaling Technology). After incubated with the secondary antibodies (anti-rabbit, 1:5000, A0545, Sigma; anti-mouse, 1:5000, A9309, Sigma), immunoreaction was visualized with an ECL detection kit (NEL104001EA, PerkinElmer Life), then quantified with the ImageJ software.

### Cell culture

H1 human embryonic stem cells (hESCs) (WiCell) were maintained in E8 medium (05940/05990, Stem Cell Technologies) on matrigel-coated dishes (354277, Corning). Cells were passaged with 0.5 mM EDTA when reached 70–80% confluency, with the presence of 5 µM Rho-associated protein kinase inhibitor Y27632 (S1049, Selleck) to improve cell viability.

Human umbilical vein endothelial cells (hECs) were purchased from Lonza (CC-2517) and maintained in endothelial medium (CC-3162, Lonza). Human adult cardiac fibroblast cells (hCFs) were purchased from Sciencell (#6330) and maintained in high glucose DMEM (C11995500CP, Gibco) supplemented with 10% fetal bovine serum (FBS).

### Differentiation of hESCs into cardiomyocytes

Cardiomyocyte induction of hESCs was performed as described previously^32^. When the hESC confluence reached 70–80% 2–3 days after plating, culture media was replaced to the differentiation basal medium (DMEM/F12 (C11330500BT, Gibco) supplemented with 19.4 μg/ml Insulin (91077 C, Sigma), 10.7 μg/ml Transferrin (T0065, Sigma), 71 μg/ml Vitamin C (A8960, Sigma), 14 ng/ml Sodium Selenite (S5261, Sigma), and 1 × Chemical Defined Lipid Concentrate (11905031, Gibco)). This day was defined as day 0. From day 0 to day 1, CHIR99021 (6 μM, S1263, Selleck) was added. IWP2 (3 μM, S7085, Selleck) was added from day 2 to day 5. Heparin (3 μg/ml, S1346, Selleck) was added from day 2 to day 7. Insulin (20 μg/ml, 91077 C, Sigma) was added to maintain hESC-derived cardiomyocytes (hCMs) from day 7 onward. Beating clusters of hCMs were normally observed at day 7. At day 11 to 13, the hCMs were further metabolically purified by using glucose-free DMEM (11966-025, Gibco) supplemented with 20 mM lactate (L7022, Sigma)^33^.

### Differentiation of hESCs into vascular smooth muscle cells

Differentiation of hESCs into vascular smooth muscle cells (hSMCs) was performed as described previously with minor modifications^34^. 1 day before differentiation, hESCs were dissociated with 0.5 mM EDTA and plated onto matrigel-coated dishes at a density of 5 × 10^4^ cells/cm^2^ in E8 medium. Upon differentiation, the medium was changed to differentiation basal medium mentioned above. From day 0 to day 1.5, cells were treated with 20 ng/ml bFGF (AF-100-18B, Peprotech), 10 μM LY294002, and 10 ng/ml BMP4. From day 1.5 to day 5, cells were treated with 20 ng/ml bFGF and 50 ng/ml BMP4 (314-BP/CF, R&D). Medium was renewed at day 3.5. At day 5, cells were dissociated into single cells by Accutase (7920, Stem cells Technology), and then seeded onto gelatin-coated plates at a density of 2 × 10^4^/cm^2^. Cells were then treated with 10 ng/ml PDGF-BB (100-14B-10, PeproTech) and 2 ng/ml TGF-β1 (240-B/CF, R&D) for at least another 12 days. Half medium renewal was performed every 2 to 3 days. Confluent cells were passaged onto gelatin-coated plates at a ratio of 1:2. Differentiated hSMCs were maintained in high glucose DMEM supplemented with 10% FBS after 12 days of PDGF-BB and TGF-β1 treatment.

### Isolation and culture of primary cardiomyocytes

For isolation of arCMs, hearts were digested using a Langendorff system in perfusion buffer (130 mM NaCl, 5 mM KCl, 1 mM MgCl_2_, 0.5 mM NaH_2_PO_4_, 10 mM HEPES, 10 mM glucose, 10 mM 2,3-butanedione monoxime, 10 mM taurine, at pH 7.5) with 1 mg/ml collagenase II (LS004177, Worthington). The left ventricle became soft and flaccidness after an ~20-min digestion. Cardiomyocytes from the left ventricle were then isolated by gravity three times and then plated onto laminin-coated culture dishes at a density of 1×10^4^ cells/cm^2^. Adult cardiomyocytes were cultured in M199 (M7528, Sigma) supplemented with 5 mM creatine (C-0780, Sigma), 5 mM taurine (T-7146, Sigma), 2 mM L-carnitine (C-0283, Sigma) and 1% penicillin–streptomycin.

For isolation of neonatal rat cardiomyocytes (nrCMs), ventricles of the hearts were removed from 1-day-old Sprague Dawley rats, and minced, followed by complete digestion with 1 mg/ml collagenase II (LS004177, Worthington) and 0.125% trypsin. nrCMs were separated from fibroblasts by differential plating and then cultured in gelatin-coated tissue culture plates at a density of 2 × 10^4^ cells/cm^2^ in medium containing DMEM/F12, 10% FBS, 1% penicillin–streptomycin, and 1% L-glutamine.

### Cytotoxicity assay

hCMs, hSMCs, hECs, and hCFs were dissociated with 0.25% trypsin/EDTA into single cells and then plated onto 96-well plate at a density of 6 × 10^3^ cells/cm^2^, 1.5 × 10^3^ cells/cm^2^, 3 × 10^3^ cells/cm^2^, and 1.5 × 10^3^ cells/cm^2^, respectively. Plating of primary nrCMs and arCMs was described above. After 24 hours of culture, cells were infected with the indicated dosage of AdSpike or AdVector adenovirus. 2 hours later, culture medium containing the virus was replaced with fresh culture medium. 48 hours after infection, cells were subjected to hypoxia-reoxygenation treatment. Then the cell viability was determined by a standard calcein-AM/PI/Hoechst cytotoxicity assay and quantified by the Operetta CLS high-content analyses system (PerkinElmer Life).

### Hypoxia-reoxygenation modeling for monolayer cultured cells

The hypoxia-reoxygenation modeling was performed in a hypoxia chamber with 1% O_2_ and then moved back to normal cell incubator. For hypoxia, cells were cultured in the chamber in serum-free and glucose-free medium for 12 hours (for hCMs, hSMCs, hECs, and hCFs) or 6 hours (for nrCMs). For hypoxia of arCMs, cells were cultured in the hypoxia buffer (125 mM NaCl, 8 mM KCl, 1.25 mM MgSO_4_, 1.2 mM KH_2_PO_4_, 20 mM HEPES, 5 mM sodium lactate and 1 M CaCl_2_, pH 6.6) and treated with 1% O_2_ for 1 hour.

### Ca^2+^ measurement under electrical field stimulation

Isolated cardiomyocytes were seeded on matrigel-coated glass coverslip and loaded with 1 μM Fura-2 AM (F1221, Invitrogen) in Tyrode’s buffer (for hCMs and nrCMs: 140 mM NaCl, 5 mM KCl, 2 mM MgCl_2_, 10 mM HEPES, 10 mM glucose, 1.8 mM CaCl_2_, pH 7.4; for arCMs:129 mM NaCl, 4 mM KCl, 23.8 mM NaHCO_3_, 0.5 mM MgSO_4_, 0.9 mM NaH_2_PO_4_, 11 mM glucose, 1.8 mM CaCl_2_, pH 7.4) at 37 °C for 15 minutes. Cells were then washed with Tyrode’s buffer and applied electrical field stimulation at increasing frequencies in a perfusion chamber using the IonOptix System (IonOptix^®^). Ca^2+^ transients were recorded with 40× objective and all parameters were calculated offline using the IonWizard 6.3.4 software.

### Immunostaining

Cells or tissue slices were fixed in 4% paraformaldehyde for 10 minutes, permeabilized with 0.4% (vol/vol) Triton X-100 for 10 minutes, and blocked with 5% bovine serum albumin for 1 hour at room temperature. Then cells or slices were incubated with primary antibody at 4 °C overnight followed by the secondary antibodies for 1 hour at room temperature. The following primary antibodies were used: α-actinin (A7811, Sigma), cTnT (MA512960, ThermoFisher), SM22α (ab14106, Abcam), ACE2 (ab15348, Abcam), CD31 (ab28364, Abcam), and α-SMA (BM0002, Boster). Images were taken using a Zeiss Cell confocal laser scanning microscope 800 (Zeiss).

### Terminal deoxynucleotidyl transferase-mediated dUTP Nick End Labeling (TUNEL) assay

Cell apoptosis was determined using TUNEL BrightRed Apoptosis Detection Kit (Vazyme Biotech, A113-01) according to manufacturer’s instructions. In brief, cells were fixed in 4% PFA for 15 minutes and then permeabilized with 0.2% (vol/vol) Triton X-100 for 5 minutes at room temperature. Cells were then incubated with the TUNEL label reaction solution for 60 minutes at 37 °C. Images were captured and quantified by the Operetta CLS high-content analyses system (PerkinElmer Life).

### Human engineered heart tissue (hEHT) fabrication and adenovirus infection

To generate aligned 3D human cardiac tissue bundle, 14 × 12 mm^2^ polydimethylsiloxane (PDMS, SYLGARD184, Dow Corning) molds with Velcro frame (12 mm long) were used. hEHTs were fabricated and cultured as shown before^18^. Hydrogel solution contains 1 × 10^6^ cells per 120 μl volume. For adenovirus infection, concentrated virus was directly added to the hydrogel solution and the MOI was calculated according to the total cell number.

### Hypoxia-reoxygenation for hEHTs

A hypoxic chamber (27310, STEMCELL) was used to simulate anoxic environment on day 8. In the hypoxic environment (1% O_2_), hEHTs were cultured in glucose-free DMEM without FBS. After 6 hours, hEHTs were transferred to normoxia environment.

### hEHT contractility test

hEHTs were paced at 1.5 Hz by the programmable electrical stimulator (YC-2, Chengdu Instrument Factory) in 10 V electric field stimulation. Video analysis of the hEHT contraction: videos were recorded with the frame rate of 25 fps, and the plugin MYOCYTER was used to analyze the amplitude, peak time 50%, and contraction frequency of the hEHTs in the video. Assessment of contractile force in stepwise stretching: hEHTs were loaded into the customized mechanical test system with temperature control, electric field stimulator, and force transducer on day 24. The recordings were generated in Tyrode’s solution with 1.8 mM Ca^2+^ in 0%-8% stretch with 2% per step. The mechanical stretch data of hEHTs was analyzed by MATLAB.

### Statistics and reproducibility

All data are presented as mean ± SEM. Statistical analyses were performed with GraphPad Prism software (version 8.0.2). The number of samples and the specific statistical hypothesis testing method (unpaired, two-tailed Student’s *t* test or Steel-Dwass test, one- or two-way ANOVA with Bonferroni post hoc test, bivariate linear regression analysis) for each comparison are described in the legends of the corresponding figures. *P* < 0.05 was considered statistically significant.

### Reporting summary

Further information on research design is available in the Nature Research Reporting Summary linked to this article.

## Supplementary information

Supplementary Information

Description of Additional Supplementary Data

Supplementary Data 1

Reporting Summary

## Data Availability

The source data behind the graphs can be found in Supplementary Data 1. Uncropped blots are available in Supplementary Fig. 6. All other data and resources are available from the corresponding authors on reasonable request.
