# Supplementary material for: Spike-based adenovirus vectored COVID-19 vaccine does not aggravate heart damage after ischemic injury in mice
Source: Commun Biol. 2022 Sep 2;5:902. doi: 10.1038/s42003-022-03875-y (PMC9439278; doi:10.1038/s42003-022-03875-y)
Supplement: Supplementary file 1 — Supplementary Information [file 42003_2022_3875_MOESM1_ESM.pdf]

Supplementary Information

Spike-based adenovirus vectored COVID-19 vaccine does not aggravate heart damage after ischemic injury in mice

Shanshan Gu, Zhongyan Chen, Xiangfu Men, Ge Liu, He Xu, Liying Huan, Linwei Wu, Jixing Gong, Ding Chen, Bingqing Xue, Lihang Zhu, Zhongjun Wan, Jianqing Lin, Xiaolong Cai, Xiaoyan Zhang, Jia Wang, Donghui Zhang and Nan Cao

The supplementary information includes:

Supplementary Figure 1 to 6

Supplementary Figure 1

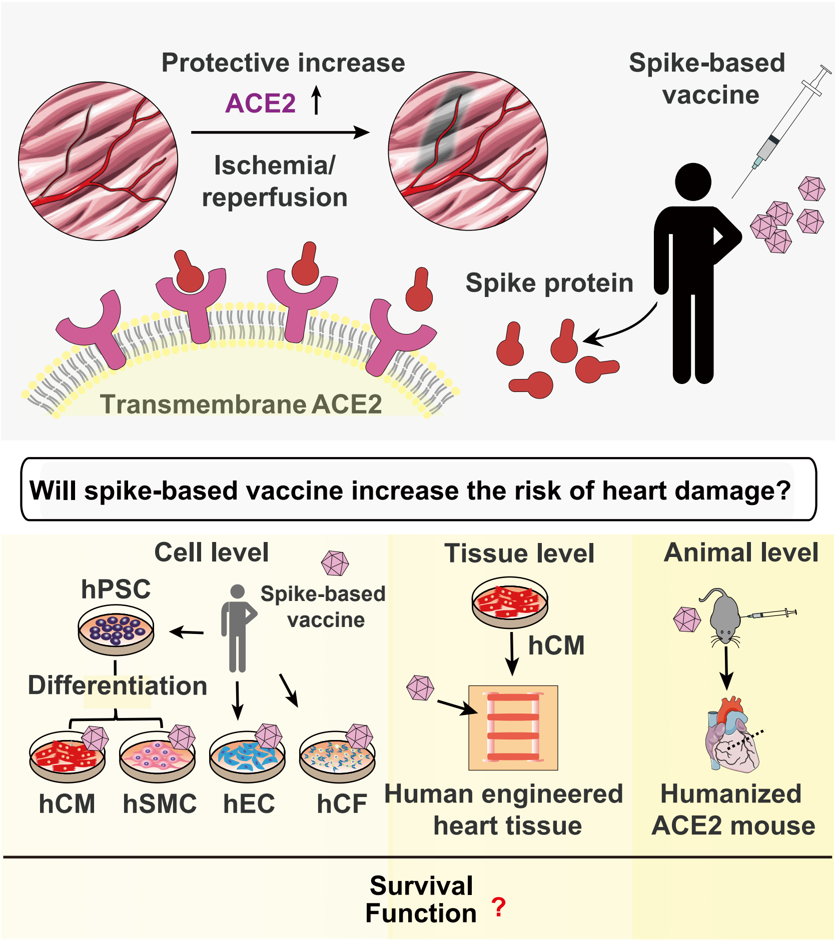

Supplementary Figure 1. Rationale and strategy of this study for evaluating the effects of spike-based vaccine on the ischemic heart.

Supplementary Figure 2

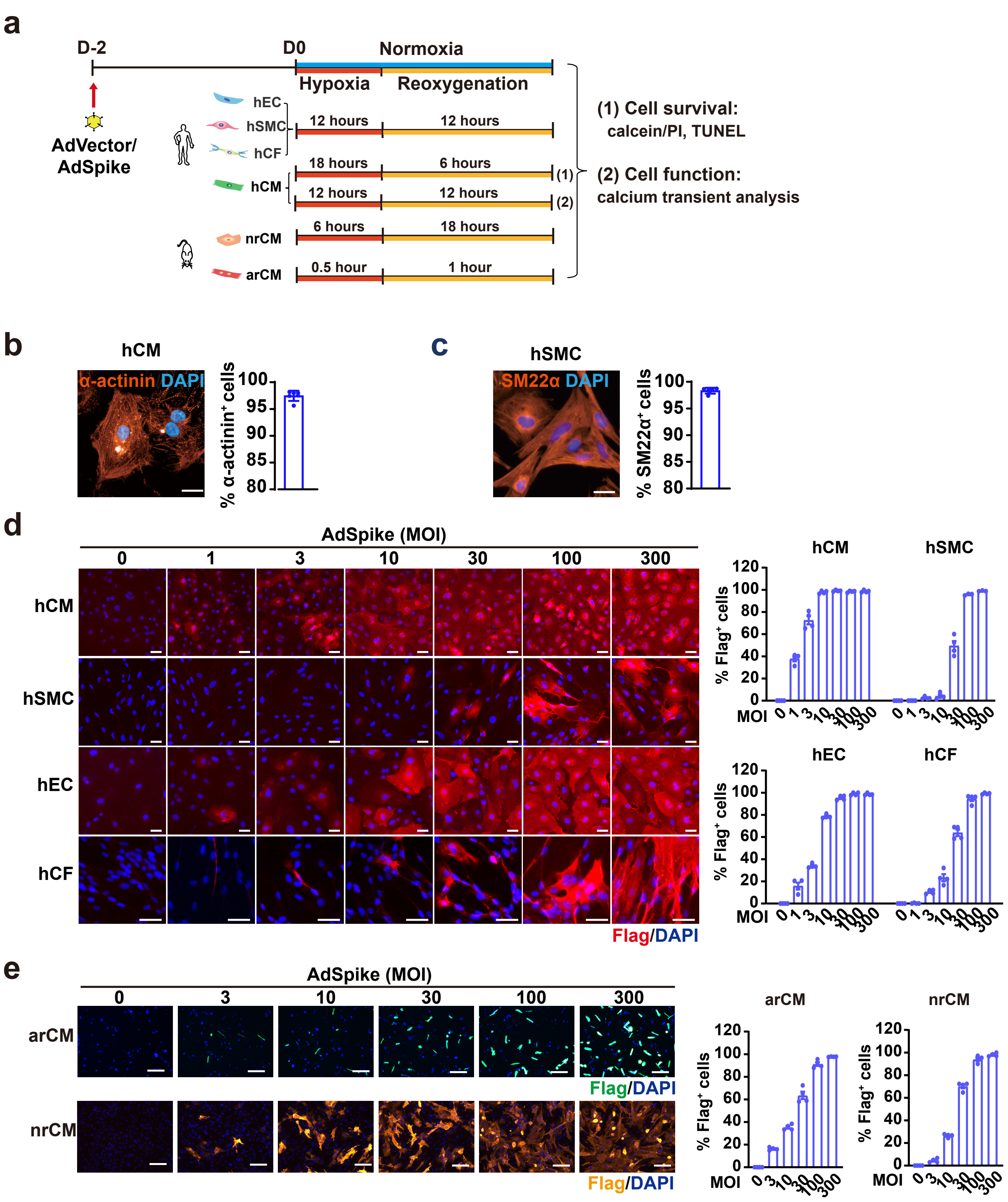

Supplementary Figure 2. The infection of AdSpike in cardiac cells. a Schematic for *ex vivo* assessment of the effect of AdSpike on cardiac cells with or without hypoxia-reoxygenation (hyp-reox) injury. hCF, human cardiac fibroblast; hCM, human cardiomyocyte; hEC, human endothelial cell; hSMC, human smooth muscle cell; nrCM, neonatal rat cardiomyocyte; arCM, adult rat cardiomyocyte. **b-c** Representative and quantitative immunostaining analysis of α-actinin⁺ hCMs (**b**) and SM22α⁺ hSMCs (**c**). n=5-6 for each group. Scale bar, 20 μm. **d** Representative immunostaining analysis of spike expression with quantification of the spike-flag⁺ cells in hCMs, hSMCs, hECs, hCFs infected with AdSpike at different MOI (multiplicity of infection). n=4 for each group. Scale bar, 50 μm. **e** Representative immunostaining analysis of spike expression with quantification of the spike-flag⁺ cells in arCMs and nrCMs infected with AdSpike at various MOI. n=4 for each group. Scale bar, 200 μm.

Supplementary Figure 3

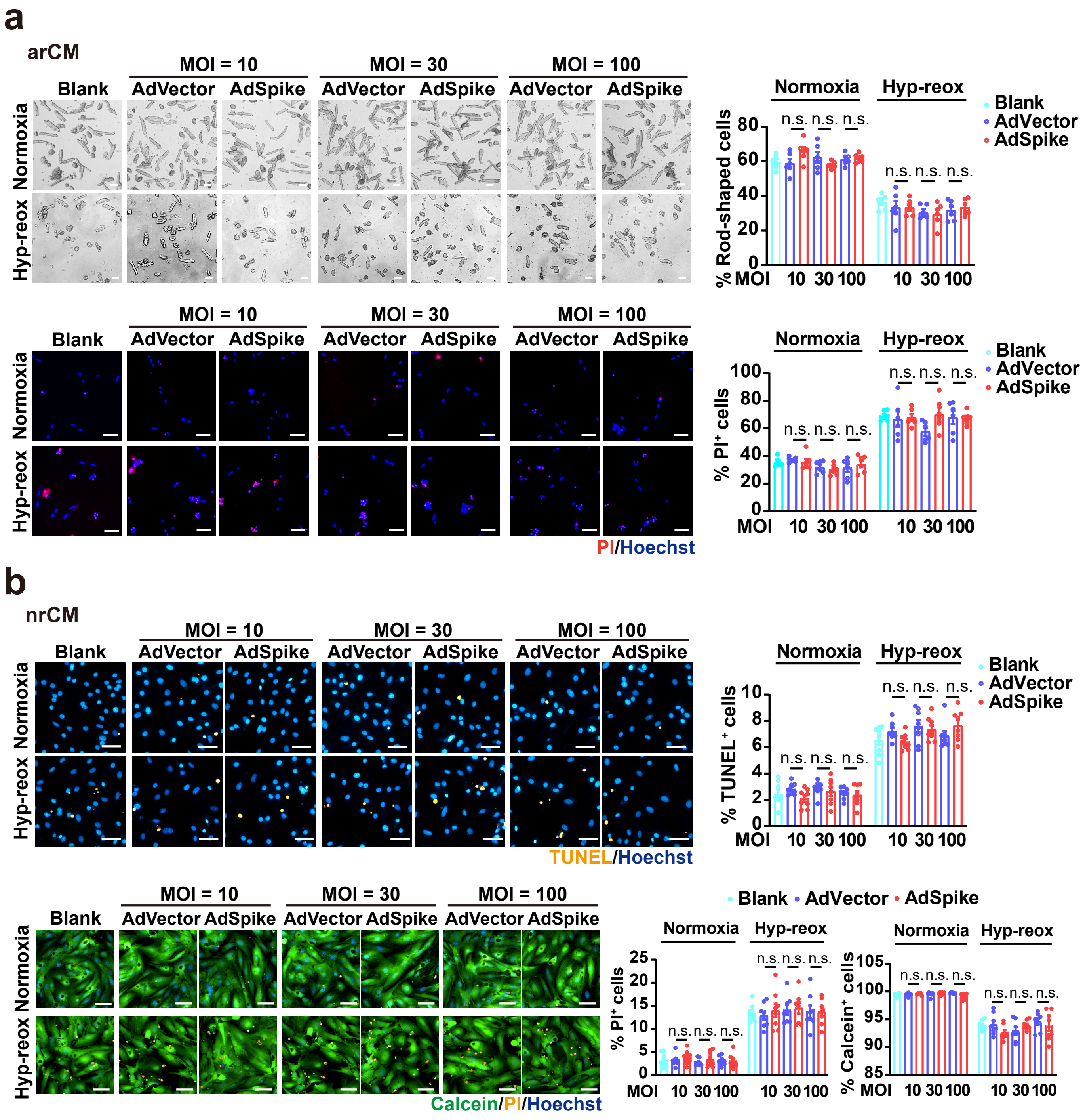

Supplementary Figure 3. AdSpike has little effects on cell survival of primary rat cardiomyocytes with or without hyp-reox injury. a Representative morphology and PI staining analysis of arCMs infected by AdVector or AdSpike with or without hyp-reox injury, with quantification of the rod-shaped healthy cells and PI⁺ apoptotic cells. n=6 for each group. Scale bar, 50 μm. **b** Representative staining images with quantification of TUNEL⁺, PI⁺, or calcein⁺ nrCMs infected by AdVector or AdSpike with or without hyp-reox injury. Scale bar, 50 μm. n=8 for each group. Statistical significance was assessed using the 2-way ANOVA with Turkey post-test. Data are presented as mean ± SEM; n.s., not significant.

Supplementary Figure 4

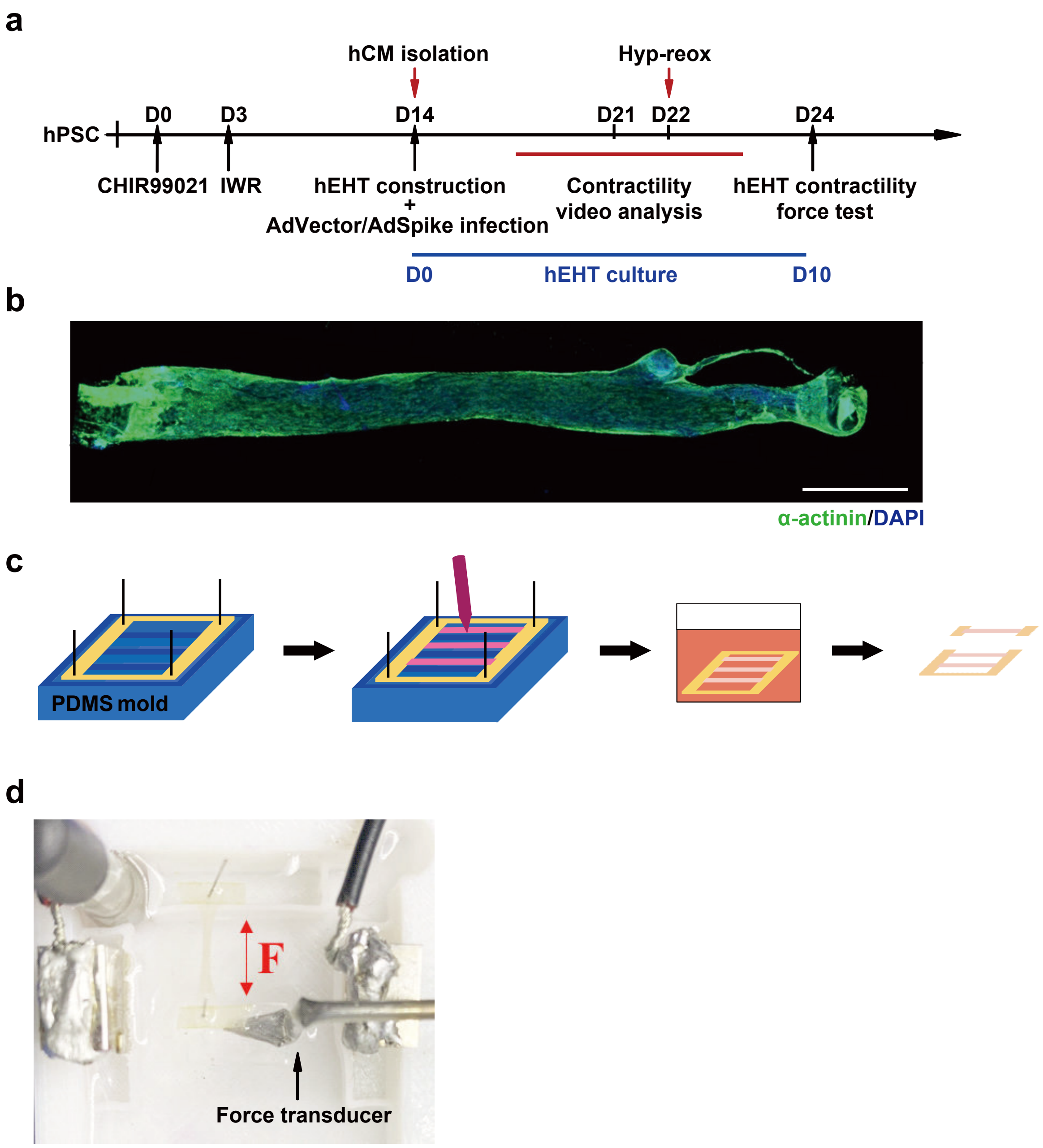

Supplementary Figure 4. The principle of the human engineered heart tissue (hEHT) production and the steps of functional test. a The timeline of the assay based on the hEHT model, including the hPSC (human pluripotent stem cell) differentiation into cardiomyocytes (CM), hEHT construction, AdVector/AdSpike infection, contractility video analysis, and contractility force test. **b** Multi stitching immunofluorescence image of the whole hEHT bundle. Cardiomyocytes are labeled with anti-α-actinin (green) and nucleus with DAPI (blue). Scale bar, 1 mm. **c** Schematic diagram of hEHT construction. **d** The equipment image of mechanical contractility force test for hEHTs.

Supplementary Figure 5

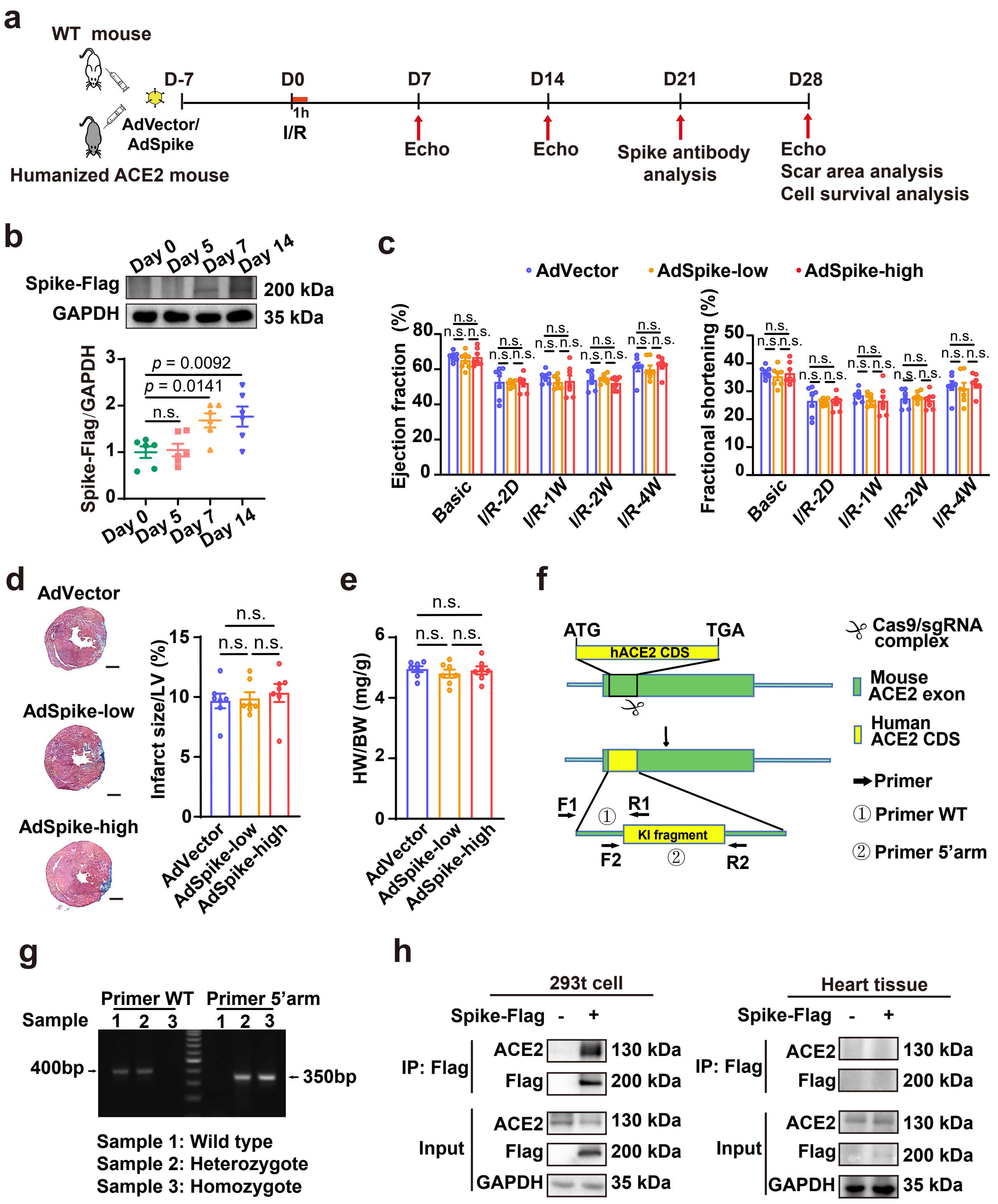

Supplementary Figure 5. AdSpike has little effects on heart damage repair after myocardial I/R on wild-type (WT) mice *in vivo*. a Schematic for evaluating the effect of AdSpike in ischemic heart *in vivo*. I/R, ischemia/reperfusion; Echo, echocardiography. **b** The protein level of Flag-fused spike in the thigh muscle where the vaccine is injected at different timepoints post-vaccination. n=6 for each group **c** Ejection fraction and fractional shortening of WT mouse hearts at various time points post-I/R measured by echocardiography. D, day; W, week. n=7 for each group. **d** Masson-Trichrome staining of heart cross sections of WT mice 4 weeks post-I/R (left) with quantification of scar size (right). n=7 for each group. Scale bar, 1 mm. **e** Quantification of heart weight (HW) relative to body weight (BW) of WT mice at 4 weeks post-I/R. n=7 for each group. **f** Strategy for generating humanized ACE2 (hACE2) mouse model. **g** Genotyping of hACE2 mouse. **h** Co-immunoprecipitation assay using Flag antibody in 293T cells (left) and heart tissue (right) with or without Flag-tagged spike overexpression. Statistical significance was assessed using the 2-way ANOVA with Turkey post-test (**c**) or the one-way ANOVA with Turkey post-test (**d** and **f**). Data are presented as mean ± SEM; n.s., not significant.

Supplementary Figure 6

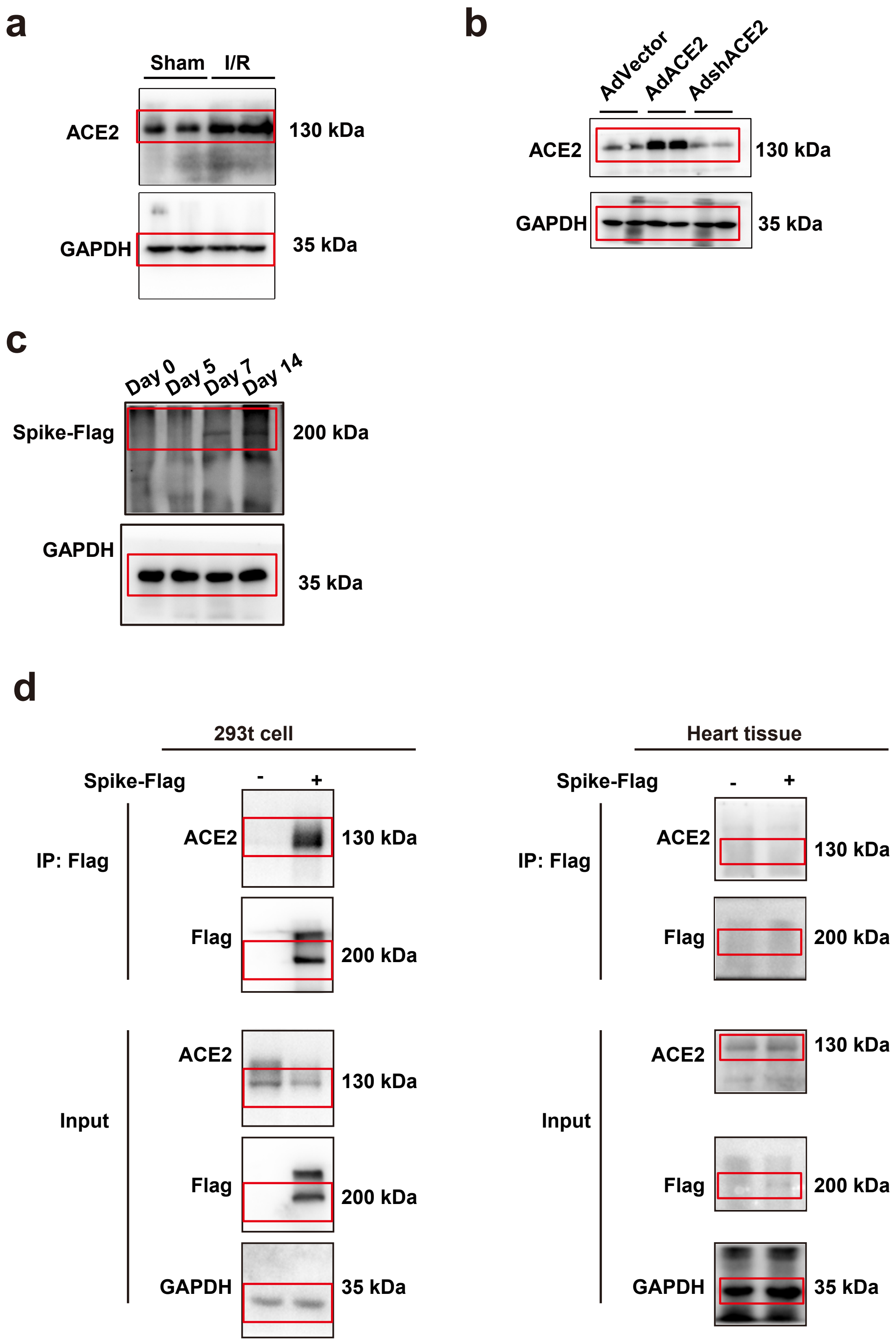

Supplementary Figure 6. Uncropped and unedited blot images. The images in the red box were edited and presented in Fiure 1a (**a**), Figure 1c (**b**), Supplementary Figure 5b (**c**) and Supplementary Figure 5h (**d**).
