# Supplementary material for: Spike-based adenovirus vectored COVID-19 vaccine does not aggravate heart damage after ischemic injury in mice
Source: Commun Biol. 2022 Sep 2;5:902. doi: 10.1038/s42003-022-03875-y (PMC9439278; doi:10.1038/s42003-022-03875-y)
Supplement: Supplementary file 2 — Description of Additional Supplementary Data [file 42003_2022_3875_MOESM2_ESM.pdf]

Description of Additional Supplementary Files

File name: Supplementary Data 1

Description: The source data behind the graphs in the paper
