# Supplementary material for: Spike-based adenovirus vectored COVID-19 vaccine does not aggravate heart damage after ischemic injury in mice
Source: Commun Biol. 2022 Sep 2;5:902. doi: 10.1038/s42003-022-03875-y (PMC9439278; doi:10.1038/s42003-022-03875-y)
Supplement: Supplementary file 4 — Reporting Summary [file 42003_2022_3875_MOESM4_ESM.pdf]

nature portfolio

Corresponding author(s): Nan Cao

Last updated by author(s): Aug 16, 2022

Reporting Summary

Nature Portfolio wishes to improve the reproducibility of the work that we publish. This form provides structure for consistency and transparency in reporting. For further information on Nature Portfolio policies, see our Editorial Policies and the Editorial Policy Checklist.

Statistics

For all statistical analyses, confirm that the following items are present in the figure legend, table legend, main text, or Methods section.

n/a	Confirmed	
☐	☒	The exact sample size (*n*) for each experimental group/condition, given as a discrete number and unit of measurement
☐	☒	A statement on whether measurements were taken from distinct samples or whether the same sample was measured repeatedly
☐	☒	The statistical test(s) used AND whether they are one- or two-sided<br>*Only common tests should be described solely by name; describe more complex techniques in the Methods section.*
☒	☐	A description of all covariates tested
☐	☒	A description of any assumptions or corrections, such as tests of normality and adjustment for multiple comparisons
☐	☒	A full description of the statistical parameters including central tendency (e.g. means) or other basic estimates (e.g. regression coefficient) AND variation (e.g. standard deviation) or associated estimates of uncertainty (e.g. confidence intervals)
☐	☒	For null hypothesis testing, the test statistic (e.g. *F*, *t*, *r*) with confidence intervals, effect sizes, degrees of freedom and *P* value noted<br>*Give P values as exact values whenever suitable.*
☒	☐	For Bayesian analysis, information on the choice of priors and Markov chain Monte Carlo settings
☒	☐	For hierarchical and complex designs, identification of the appropriate level for tests and full reporting of outcomes
☒	☐	Estimates of effect sizes (e.g. Cohen's *d*, Pearson's *r*), indicating how they were calculated

Our web collection on statistics for biologists contains articles on many of the points above.

Software and code

Policy information about availability of computer code

Data collection	IonWizard (v6.3.4)
Data analysis	Microsoft Excel (v2013), GraphPad Prism (v8.0.2), ImageJ (v1.51j8), IDL (v8.0), IonWizard (v6.3.4), MATLAB (v2018)

For manuscripts utilizing custom algorithms or software that are central to the research but not yet described in published literature, software must be made available to editors and reviewers. We strongly encourage code deposition in a community repository (e.g. GitHub). See the Nature Portfolio guidelines for submitting code & software for further information.

Data

Policy information about availability of data

All manuscripts must include a data availability statement. This statement should provide the following information, where applicable:
- Accession codes, unique identifiers, or web links for publicly available datasets
- A description of any restrictions on data availability
- For clinical datasets or third party data, please ensure that the statement adheres to our policy

All data are available in the manuscript or the supplementary materials. Source data are provided with this paper.

Human research participants

Policy information about studies involving human research participants and Sex and Gender in Research.

Reporting on sex and gender	N/A
Population characteristics	N/A
Recruitment	N/A
Ethics oversight	N/A

Note that full information on the approval of the study protocol must also be provided in the manuscript.

Field-specific reporting

Please select the one below that is the best fit for your research. If you are not sure, read the appropriate sections before making your selection.

☒ Life sciences ☐ Behavioural & social sciences ☐ Ecological, evolutionary & environmental sciences

For a reference copy of the document with all sections, see nature.com/documents/nr-reporting-summary-flat.pdf

Life sciences study design

All studies must disclose on these points even when the disclosure is negative.

Sample size	Sample sizes were chosen on the basis of preliminary data from at least two pilot experiments and of previously published results.
Data exclusions	No data were excluded.
Replication	All in vitro and in vivo experiments were carried out with at least 3 biological replicates for each experimental group.
Randomization	Mice were assigned randomly to experimental groups.
Blinding	All the surgeries, echocardiography and analyses were conducted blind to prevent bias. For the other experiments, the researchers were not blinded to group allocation during the experiments and outcome assessment because our data analyses were based on objectively measurable data.

Reporting for specific materials, systems and methods

We require information from authors about some types of materials, experimental systems and methods used in many studies. Here, indicate whether each material, system or method listed is relevant to your study. If you are not sure if a list item applies to your research, read the appropriate section before selecting a response.

Materials & experimental systems

n/a	Involved in the study	
☐	☒	Antibodies
☐	☒	Eukaryotic cell lines
☒	☐	Palaeontology and archaeology
☐	☒	Animals and other organisms
☒	☐	Clinical data
☒	☐	Dual use research of concern

Methods

n/a	Involved in the study	
☒	☐	ChIP-seq
☒	☐	Flow cytometry
☒	☐	MRI-based neuroimaging

Antibodies

Antibodies used	α-actinin (Clone:EA-53, Cat# A7811, Sigma, 1:500)<br>cTnT (Clone:13-11, Cat# MA512960, ThermoFisher,1:500)<br>SM22α (Clone: Ploycloncal, Cat# ab14106, Abcam, 1:200)<br>ACE2 (Clone: Ploycloncal, Cat# ab15348, Abcam, 1:200 for immunofluorescence, 1:1000 for Western Blot)<br>CD31 (Clone: Ploycloncal, Cat# ab28346, Abcam, 1:200)<br>α-SMA (Clone: 1A4, Cat# BM0002, Boster, 1:200)

	GAPDH (Clone:D4C6R, Cat# 97166, Cell Signaling Technology, 1:8000),<br>Secondary antibodies conjugate peroxidase (anti-rabbit, A0545, Sigma, 1:5,000; anti-mouse, A9309, Sigma, 1:5,000,),<br>Secondary antibodies conjugated with Alexa-488 or Alexa-555 (Cat# A11001, A211121, A21135, A11008; Thermofisher; 1:500).
Validation	Antibody validation is indicated on the manufacture's website (cell images) and/or provided by data in the manuscript.

Eukaryotic cell lines

Policy information about cell lines and Sex and Gender in Research

Cell line source(s)	HEK 293 cells (thermo), human embryonic stem cell line H1 (WiCell), Human umbilical vein endothelial cells (Lonza) and Human adult cardiac fibroblast cells (Sciencell).
Authentication	Authentication of the cell lines was offered by the manufacturer or provider.
Mycoplasma contamination	Mycoplasma contamination was routinely monitored every week in our laboratory. Cells were confirmed for the absence of mycoplasma contamination before every cellular experiments.
Commonly misidentified lines<br>(See ICLAC register)	No commonly misidentified cell lines were used.

Animals and other research organisms

Policy information about studies involving animals; ARRIVE guidelines recommended for reporting animal research, and Sex and Gender in Research

Laboratory animals	Adult male C57BL/6 mice were purchased from the Laboratory Animal Center of Sun Yat-Sen University. The C57BL/6 background humanized ACE2 (hACE2) mice (T037659) were purchased from Gempharmatech Co. Ltd (Nanjing, China).
Wild animals	This study did not use wild animals.
Reporting on sex	Only male animals were used in this study to avoid the disturbance from the estrogen fluctuations in the female mice.
Field-collected samples	The study did not involve samples collected from the field.
Ethics oversight	All mouse work was done in accordance with the Guide for the Care and Use of Laboratory Animals (National Institutes of Health, Publication No. 85-23, Revised), and carried out under the supervision of the Sun Yat-sen University Animal Care and Use Committee.

Note that full information on the approval of the study protocol must also be provided in the manuscript.
